# Anatomy of the endocrine pancreas in actinopterygian fishes and its phylogenetic implications

**DOI:** 10.1038/s41598-023-49404-7

**Published:** 2023-12-15

**Authors:** Bruno Chanet, Nalani K. Schnell, Claude Guintard, Wei-Jen Chen

**Affiliations:** 1 Département Origines Et Évolution, Institut de Systématique, Evolution, Biodiversité (ISYEB) (UMR 7205 MNHN-CNRS-Sorbonne Université-EPHE), Muséum National d’Histoire Naturelle, CP 30, 57 Rue Cuvier, 75005 Paris, France; 2 Institut Systématique Evolution Biodiversité (ISYEB), Muséum National d’Histoire Naturelle, CNRS, Sorbonne Université, EPHE, Station Marine de Concarneau, Place de La Croix, 29900 Concarneau, France; 3 Laboratoire d’Anatomie Comparée, ONIRIS – Ecole Nationale Vétérinaire de l’Agroalimentaire et de l’Alimentation, Nantes Atlantique, Route de Gachet, CS 40 706, 44307 Nantes Cedex 03, France; 4 Institute of Oceanography, National Taiwan University, No.1 Sec. 4, Roosevelt Road, Taipei, 10617 Taiwan

**Keywords:** Ichthyology, Phylogenetics

## Abstract

The anatomy and organisation of the endocrine pancreas in ray-finned fishes vary widely. The two main morphoanatomical character states are diffuse versus compact pancreatic tissue. The latter are called Brockmann Bodies (BBs), or principal islets. The present study is the first comprehensive survey on the anatomy of the endocrine pancreas (diffuse versus compact) across 322 actinopterygian species in 39 orders and 135 families based on literature, specimen dissections, and Magnetic Resonance Imaging (MRI). The data obtained show that large endocrine pancreatic islets (BB) have appeared several times in teleost evolution: in some ostariophysian clades and within the Salmoniformes and Neoteleostei. Acanthomorpha (spiny-rayed fishes) is the largest clade of the Neoteleostei. Within this clade, an absence of BBs is only observed in flying fishes (Exocoetidae), parrotfishes (Scarinae), and some of the scarine relatives, the Labridae. The presence of BBs in examined jellynose fish species from the Ateleopodiformes indicates support for its sister-group relationship to the Ctenosquamata (Myctophiformes + Acanthomorpha). More investigations are still needed to corroborate the presence or absence of BBs as a putative synapomorphy for a clade comprising Ateleopodiformes and Ctenosquamata.

## Introduction

In vertebrates the regulation of the glucose level in blood (glycemia) is mainly controlled by hormones (insulin and glucagon) produced by the endocrine pancreas^1,2^. Anatomically, the endocrine cells of the pancreas form islets, called the islets of Langerhans, that are generally scattered within pancreatic tissues^1,3^. In ray-finned fishes (Actinopterygii), these tissues (endocrine and exocrine pancreas) are rather diffused between the mesenteries and the coils of the intestine^1,3,4^; they may even enter the liver to varying degrees around the portal veins, forming the hepatopancreas^5,6^. In some groups, the endocrine islets agglomerate to form a few macroscopic islets called principal islets, or Brockmann bodies (BBs)^2,7^. They are often embedded in the exocrine pancreatic tissue between the hepatic lobes, close to the gallbladder, the pyloric caeca, and the spleen^8^. These macroscopic islets (BBs) can be detected through classical dissections. They are pinkish to reddish rounded nodules and look like “small peas”^9^ (p. 157) or “sesame seeds”^10^ (p. 74) at the surface of the stomach. The diameter of a BB ranges from few millimetres (mm)^8,11^ up to 10–15 mm in some species like the ocean sunfish (*Mola mola*, Molidae)^9^, lumpfish (*Cyclopterus lumpus*, Cyclopteridae)^12^, common perch (*Perca fluviatilis*, Percidae)^13^, and European anglerfish (*Lophius piscatorius*, Lophiidae)^14^. If present, there are usually one to several distinguishable Brockmann bodies^7,12^. For example, there are three BBs in the Atlantic wolffish (*Anarhichas lupus*, Anarhichadidae) and ten to fifteen BBs in the Nile tilapia (*Oreochromis niloticus*, Cichlidae) forming a “BB region”^15^.

The study of the endocrine pancreas in fishes has been and is still a focal subject area in comparative endocrinology^2^. The islets of Langerhans were first identified in fishes in the 19th Century^9,16–19^, and the first extractions of insulin conducted in different fish species as early as in the 1920’s^15,20,21^, even for clinical purposes in the 1940s in Germany, Japan, and Canada^14,22^. The large islets (BBs) in some fishes are subject to experiments in xenotransplantation^14,23^. Nevertheless, the data on the anatomy of the endocrine pancreas have rarely been taken into account in a phylogenetic context. The presence of BBs was often considered either a shared character for teleost fishes or a character only present in “higher teleosts”^3,24–26^, based on only a few observations and data. Epple and Brinn^3^ identified the presence of BBs in several teleost species and considered them a shared character for the higher teleost fish clade, the Ctenosquamata (sensu Rosen^27^) (Myctophiformes plus Acanthomorpha). However, a closer examination of the list of species examined by these authors shows that they only examined a few acanthomorph species. Accordingly, the presence of the BB cannot be considered as a putative synapomorphy of the group based on their limited data and without an outgroup comparison. The purpose of this study was to review the data published on this subject and fill in its gaps with new data acquired during this study in order to highlight the possible phylogenetic significance of the anatomy of the endocrine pancreas. We demonstrate the distribution of BBs in the phylogenetic context^28–31^ and further assess the presence/absence of BBs as a possible synapomorphy for some teleost fish clades.

## Results

**Figure 1:**
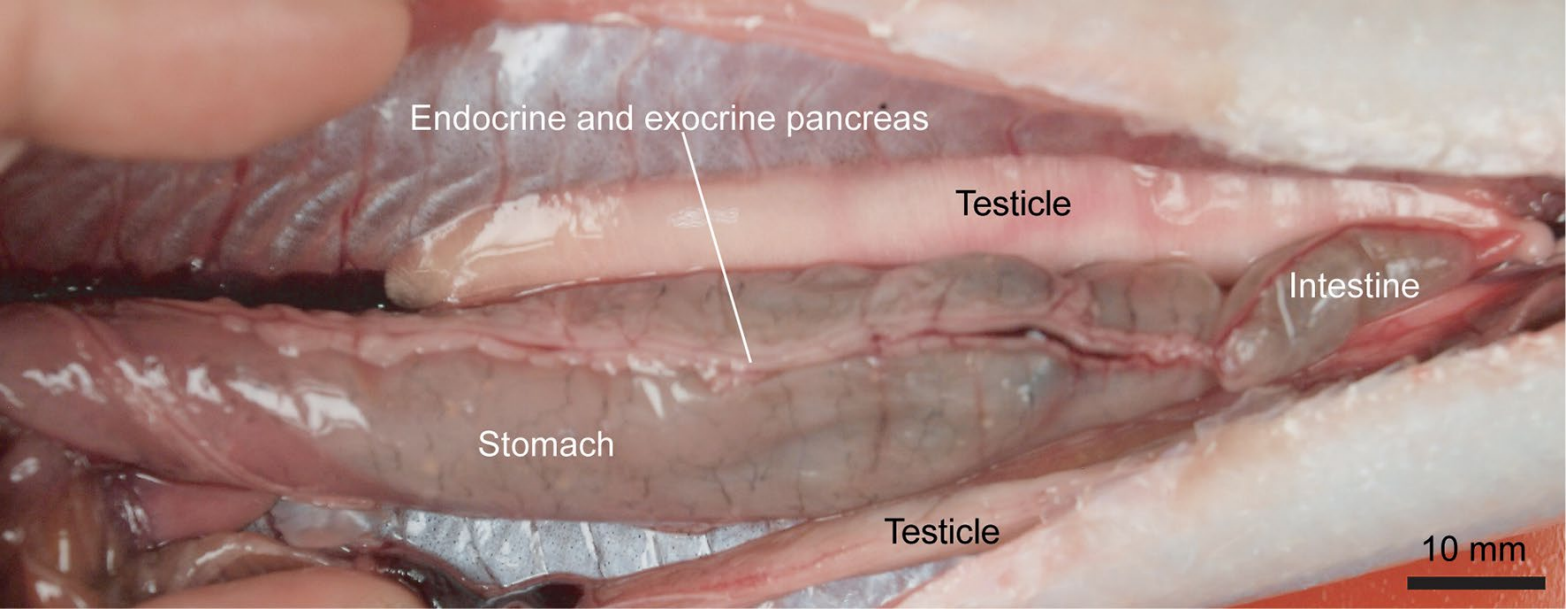
Gross internal organization and absence of the Brockmann Body (BB) in a freshly dissected specimen of *Saurida elongata*, NTUM14376, 288 mm SL. The endocrine and exocrine pancreas are embedded in fatty tissue. Anterior facing left.

**Figure 2:**
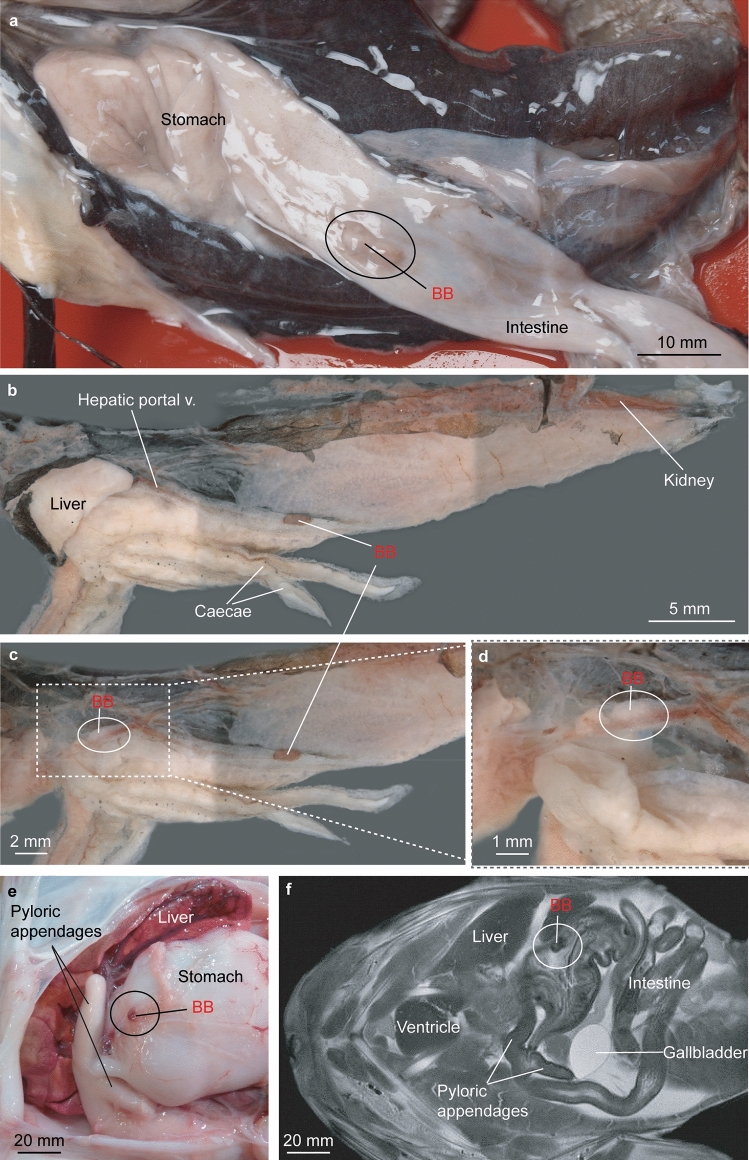
Gross internal organization and presence of the Brockmann Body (BB) in freshly dissected specimens of different teleost taxa. Anterior facing left. (**a**) *Ateleopus japonicus*, NTUM15935, 515 mm SL. (**b**–**d**) *Gymnoscopelus braueri*, MNHN 2022–0027, 117 mm SL. (**b**) posterior brown-reddish BB visible, anterior BB overlaid by the liver. (**c**) liver removed. (**d**) Zoom onto the anterior whitish BB. (**e**, **f**) *Lophius piscatorius,* MHNNT uncat. 57 mm SL. (**e**) BB in a fresh dissected specimen and (**f**) BB in an MRI image (modified from Chanet et al*.*^31^).

Within the Actinopterygii, two anatomical patterns of the endocrine pancreas were identified: diffuse (Fig. 1) versus compact pancreatic tissues (Fig. 2), or in other words, absence or presence of BBs (see also Supplementary Table 1 for a complete list of taxa). We gathered data from 194 acanthomorph species representing all the major lineages. However, so far, no data on the anatomy of the endocrine pancreas (presence or absence of at least one BB) is available for Gymnotiformes (species found only in Neotropical and South America), Lepidogalaxiiformes (only one species, *Lepidogalaxias salamandroides*, is present), Percopsiformes (species found only in North America), and Polymixiiformes (deep-sea fishes).

**Figure 3:**
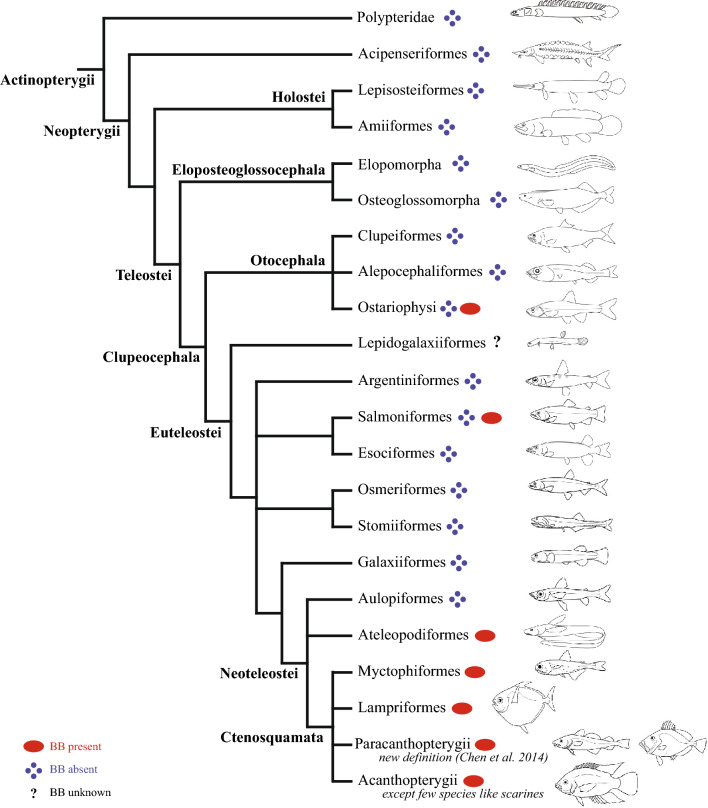
Presence and absence of BB plotted onto the molecule-based phylogeny of Actinopterygii^28^ modified after Chen et al.^29^, Dornburg & Near ^30^, and Parey et al*.*^31^. The uncertain inter-relationships are collapsed and presented as unresolved polytomies. The illustration of fishes is redrawn by JNC, YAC, and LHC (see acknowledgement) after Nelson et al*.*^41^.

*Pattern 1*. The endocrine pancreas consists of scattered microscopic islets of Langerhans within the exocrine pancreatic tissues (Fig. 1, e.g., *Saurida elongata*). This is the general condition found in actinopterygians (Supplementary Table 1). It is found in all basal actinopterygian families (Polypteridae, Acipenseridae, Polyodontidae, Lepisosteidae, and Amiidae) as well as in the teleost groups Elopomorpha, Osteoglossomorpha, Clupeomorpha, Alepocephaliformes, Argentiniformes, Esociformes, Stomiiformes, Osmeriformes, Galaxiidae, and Aulopiformes. Further, it is also found in some siluriform taxa, a few cypriniform taxa, a few salmoniform taxa, and a few families and subfamilies of acanthomorph fishes (e.g., Belonidae, Exocoetidae, Fundulidae, Scarinae, and some labrid species) (Fig. 3).

*Pattern 2*. Microscopic islets of endocrine pancreas are present along with giant islets, called principal islets, or Brockmann bodies (BBs), in the exocrine pancreatic tissues (Fig. 2A–F). The presence of at least one BB has been described for the Characiformes, a few siluriform taxa, some cypriniform taxa, some salmoniform taxa, and most acanthomorph taxa (Supplementary Table 1). We report its presence for the first time in two species of the Ateleopodiformes (*Ateleopus japonicus* and *A. purpureus*), in one species of the Myctophiformes (*Gymnoscopelus braueri*), and in most of the newly examined acanthomoph species (Supplementary Table 1 and Fig. 2A–F). Our observations show that so far only 11 acanthomorph species lack a BB (one belonid, one exocoetid, two fundulids, and seven labrids) (Fig. 3).

Finally, a special case exists in *Chanos chanos* (Chanidae, Gonorynchiformes). In this species the anatomy of the visceral region was found to be complex, without a significantly sized stomach which is followed by a very long intestine. An eventual BB could not be identified with certitude. The examinations of the anatomy of the visceral region of additional gonorynchiform species are needed to further confirm the absence of BB in this group.

## Discussion

While pancreatic islets are only known in vertebrates^26^, the origin of the anatomy of the endocrine pancreas (i.e., the presence of one or few principal islets or BBs) in ray-finned fishes has never been comprehensively explored in a broader phylogenetic context even though the presence of BBs has been documented in various teleost clades. In this study, we compile the information on presence or absence of at least one BB in different teleost fishes, provided in the literature or based on our own observations (MRIs and specimen dissections) (see Supplementary Table 1). Our results indicate that the presence of a BB has likely evolved multiple times within the Teleostei: in the Ostariophysi, in the Salmoniformes, and in the Neoteleostei e.g.,^3,7,8,10,12,16^ (Fig. 3).

The Eloposteoglossocephala is a newly constituted clade grouping the two early teleost fish lineages, Elopomorpha (tarpon, bonefish and eels) and Osteoglossomorpha (bony tongues), based on the analyses from seven high-quality new genome assemblies^31^ (Fig. 3). All the taxa included in this clade and examined show the absence of BBs (Supplementary Table 1). Since the absence of BBs is a plesiomorphic state, our results are not informative to either support or reject this new phylogenetic hypothesis.

The Ostariophysi is the second-largest superorder of fishes and includes the majority of freshwater fishes in the world^41^. Within the superorder, the presence of BBs was documented in at least some species except the Gonorynchiformes, for which the anatomy of the visceral region needs to be examined for more species (Supplementary Table 1). The data on the presence/absence of at least one BB are available from nine out of 23 currently recognized cypriniform families. Out of those, a BB is present in at least one representative species from seven cypriniform families^8,10,12,43^. The BB was documented to be absent in the Cobitidae^10^ and Xenocypridae^42^, yet only one species from each family was investigated.

The Siluriformes comprise about 35 living families^44^. The BB status has been examined for species in the Trichomycteridae^45^, Pimelodidae^46^, Ictaluridae[e.g.,^8,48^, Siluridae^43,46^, Bagridae^49^, Clariidae^50^, and Heptapteridae^51^. The presence of BBs was documented in at least one examined species of the four former families. The Characiformes contains at least 24 living families and about 2300 species^41,52^. However, only the data for two species belonging to the same family, Characidae, are available, and a BB is documented to be present^53^. More data on the presence/absence of a BB would be needed to better understand its distribution within the Characiformes.

The Salmoniformes contains a single family, Salmonidae, including salmon, trout, chars, whitefishes, and other allies. This fish order is conventionally placed within the superorder of the Protacanthopterygii, together with Esociformes and “Osmeriformes”^41,54^. Molecular results resolved a non-monophyly of the traditionally defined Protacanthopterygii and Osmeriformes^28–30^ (Fig. 3). BB presence/absence data are available for “protacanthopterygian” groups, including the Salmonidae, Esocidae (Esociformes**)**, Umbridae (Esociformes**)**, Alepocephalidae (Alepocephaliformes), Argentinidae (Argentiniformes), Osmeridae (Osmeriformes), Plecoglossidae (Osmeriformes), and Galaxiidae. The BBs are absent in all, but some salmonids^3,8,10,43^ (Fig. 3).

Jellynose fishes constitute the small fish order Ateleopodiformes, with about 12 species in the single family Ateleopodidae^40^. The phylogenetic placement of this enigmatic fish order has been the subject of considerable debate among either morphology- or molecule-based studies^30,41^. Based on morphology, Olney et al.^55^ concluded that it forms an unresolved trichotomy with the stomiiforms and eurypterigians (Aulopiformes plus Ctenosquamata). While molecular studies revealed the sister-group relationship between the Stomiiformes and Osmeriformes, the sister group of the Ateleopodiformes remained unresolved (Fig. 3). Our dissections show that a BB is present in two species of the Ateleopodiformes, but is absent in the aulopiform species (Figs. 1 and 2A; Supplementary Table 1). Based on its distribution across teleost fish taxa, we consider its presence as a synapomorphy of the fishes comprising Ateleopdiformes and Ctenosquamata (Myctophiformes plus Acanthomorpha), or in other words, the Neoteleostei exclusive of the Aulopiformes (Fig. 3). Epple and Brinn^3^ already proposed that the presence of a BB is a synapomorphy of the Ctenosquamata. Yet, this hypothesis was weakly supported as the authors examined only few ctenosquamat representatives, lacked data on myctophids, and included only five acanthomorph genera (*Gadus*, *Lophius*, *Fundulus*, *Xiphophorus,* and *Tilapia*). Moreover, they followed the classification proposed by Rosen^27^, where gadids and lophiids were members of the Paracanthopterygii and excluded from the Acanthomorpha. In spite of that, Epple and Brinn’s^3^ work remains pioneering in understanding the distribution of pancreatic character-states in vertebrates, especially in ray-finned fishes. In the present study, we aim to reconsider previously published anatomical data, concomitant with our own observations in the light of molecule-based phylogenies^28–31^. We support the suggested hypothesis from Epple and Brinn^3^ that the presence of a BB is a putative synapomorphy of the Ctenosquamata plus Ateleopodiformes.

Within the Acanthomorpha (Lampriformes, Paracanthopterygii, and Acanthopterygii), a BB has been described in a large number of species except for belonids^8^ and seven labrid species^8,19,56,57^, [this study]. Four of those labrid species without BB belong to the labrid subfamilies Scarinae, Labrinae, and Cheilinae. These three subfamilies form a moderately supported monophyletic group within the phylogenetic tree based on mitochondrial and nuclear DNA sequences^58^. The absence of a BB could be a putative synapomorphy of this clade. However, with more than 600 species, the Labridae forms a rich and diverse clade of the Acanthomorpha^58^, and the anatomy of the endocrine pancreas has only been studied in 12 labrid species prior to this study. Here, we report for the first time that the BB is absent in two species of *Cheorodon* (Hypsigenyinae) based on specimen dissections, which suggest that the absence of a BB may have occurred multiple times in the evolutionary history of the Labridae. More investigations into the visceral anatomy of the Labridae are still needed to draw a clear conclusion.

The present work is mainly supported by data compiled from the literature and shows the importance of both comparative anatomy of soft tissue and previous literature on it to enlighten the evolutionary history of vertebrates. Our understanding of the phylogeny of vertebrates has been for a long time a “bony story.” However, comparative analyses of soft tissues provide valuable data to address phylogenetic questions. Future work on the presence/absence of a BB in the Gonorynchiformes, Gymnotiformes, Lepidogalaxiformes, Percopsiformes, and Polymixiiformes would complete the present work. Further, the presented evolutionary scenarios on the endocrine pancreas are based on the untested hypothesis that BBs are homologous throughout the different taxa. Future studies on the homology of different types of endocrine cells within the BB found in non-Ctenosquamata and Ctenosquamata fishes will be of great interest.

## Materials and methods

### Ethical approval

This research was performed at the National Taiwan University (NTU) and the Muséum National d'Histoire Naturelle (MNHN) and in accordance with both institution’s guidelines regarding animal research. As this project did not involve experiments on live fish, no ethics statement was required.

### Data collection

First, more than 150 previous publications on the anatomy of the pancreas in different teleost taxa have been reviewed. As to the acquisition of new data, all the specimens examined in the present study were from museum collection material (see below). Examination of the Magnetic Resonance Imaging (MRI) images^13,32^ (MRI images available at the digital fish library[DFL] at http://www.digitalfishlibrary.org/index.php^33^) as well as dissections of specimen of 20 species (see material listed below) were carried out to further complete the data for the present investigation. In total, we compiled data from 322 teleost fish species. A similar literature-based approach has been employed by Parenti^34^ for the evaluation of the phylogenetic significance of bone types in euteleost fishes, by Wilson & Castro^35^ for investigating the “loss” of the stomach in fish species, and by Chanet & Meunier ^36^ in studying the comparative anatomy of the thyroid gland in vertebrates.

Rennie^7^ (p. 375) noticed that the BBs have a “constant occurrence in definite position,” and no polymorphism in their shape has been described or observed from dissections of specimens within the same species. Epple and Brinn^3^ reported that BBs are topographically never completely separated from the exocrine pancreas. The BBs are separated from the exocrine tissue by only a connective tissue sheet around them. However, this sheet has been reported incomplete many times^3,8,36–38^. It is noteworthy that the BB is a vascularized structure and appears to be quite fragile. Once the specimen has been preserved, BB presence is hard to assess^13^, so it is necessary to have fresh material available for dissection^7^. We made similar observations when examining numerous MRIs, available at the DFL. Once the specimen has been preserved prior to scanning, the presence of BBs can no longer be detected with certainty. In contrast, BBs have been detected easily on MRIs from fresh specimens of the European anglerfish (Fig. 2F) and common perch^13^. Some of the digital photographs were taken with a Zeiss axiocam attached to a Zeiss Discovery V20 and are composite images taken with the Z-stack option of the Zeiss Zen software in order to obtain a depth of field. Taxonomic names were verified in Eschmeyer's Catalog of Fishes^39^ available at https://www.calacademy.org/scientists/projects/eschmeyers-catalog-of-fishes. Classification of Telestei follows Dornburg & Near^30^. Identified pancreatic character patterns have been mapped on the state-of-the-art phylogeny of ray-finned fishes based on molecular data^28–31^ to show the presence or absence of the Brockmann Bodies.

### Material examined

Alepocephalidae, *Alepocephalus bicolo*r Alcock, 1891, NTUM13155 (2 specimens, 96–171 mm SL); NTUM uncat. (10 specimen, NA). Chanidae, *Chanos chanos* (Fabricius, 1775) NTUM16304 (1 specimen, ca. 400 mm SL). Argentinidae, *Argentina kagoshimae* Jordan & Snyder, 1902, NTUM15947 (4 specimen, 290–354 mm SL). Plecoglossidae, *Plecoglossus altivelis* (Temminck & Schlegel, 1846), NTUM15526 (1 specimen, 190 mm SL). Galaxiidae, *Galaxias maculatus* (Jenyns, 1842), TMAG D4022-4023 (2 spcimen, NA). Synodontidae, *Saurida elongata* (Temminck & Schlegel, 1846), NTUM14376 (1 specimen, 288 mm SL); *Saurida micropectoralis* Shindo & Yamada, 1972, NTUM15058 (3 specimen, 246–268 mm SL). Ateleopodidae, *Ateleopus japonicus* Bleeker, 1853, NTUM15936 (3 specimen, 478–560 mm SL); NTUM15935 (1 specimen, 515 mm SL). Myctophidae, *Gymnoscopelus braueri* (Lönnberg, 1905) MNHN 2022–0027 (1 specimen, 117 mm SL). Diretmidae, *Diretmoides veriginae* Kotlyar, 1987, NTUM16486 (2 specimen, 123–124 mm SL). Ophidiidae, *Ophidion barbatum* Linnaeus, 1758, MNHN 2022–0013 (1 specimen, 199 mm SL). Gempylidae, *Promethichthys prometheus* (Cuvier, 1832), NTUM16292 (2 specimen, 240–350 mm SL). Menidae, *Mene maculata* (Bloch & Schneider, 1801) NTUM uncat. (1 specimen, NA). Labridae, *Choerodon azurio* (Jordan & Snyder, 1901) NTUM16775 (1 specimen, 101 mm SL); *Choerodon schoenleinii* (Valenciennes, 1839) NTUM16297 (1 specimen, 260 mm SL); *Scarus ghobban* Forsskål, 1775, NTUM16293 (1 specimen, 316 mm SL). Lophiidae, *Lophiomus setigerus* (Vahl, 1797) NTUM uncat. (3 specimen, NA); *Lophius piscatorius* Linnaeus, 1758, MHNNT uncat. (3 specimen, 27–57 mm SL) Sciaenidae, *Chrysochir aurea* (Richardson, 1846) NTUM16732 (1 specimen, 332 mm SL). Scorpaenidae, *Scorpaenopsis neglecta* Heckel, 1837, NTUM15712 (6 specimen, 107–122 mm SL).

### Supplementary Information

Supplementary Information.

## Data Availability

All data generated or analyzed during this study are included in this published article and its Supplementary table 1.
