# Supplementary material for: Anatomy of the endocrine pancreas in actinopterygian fishes and its phylogenetic implications
Source: Sci Rep. 2023 Dec 15;13:22501. doi: 10.1038/s41598-023-49404-7 (PMC10728084; doi:10.1038/s41598-023-49404-7)
Supplement: Supplementary file 1 — Supplementary Information. [file 41598_2023_49404_MOESM1_ESM.docx]

**Supplementary table 1** List of Actinopterygii showing presence/absence of BBs. Data taken from previous literature and our own dissections and observations. Classification follows Dornburg & Near [30]. Asterisk (*) denotes tentative result from our observation; further confirmation is required. Question mark (?) indicates pancreas is described in the literature, but presence/absence of BBs is not mentioned

| **Taxon** | **species** | **BB**  **present/absent** | **reference** |
| --- | --- | --- | --- |
| **ACTINOPTERYGII** |  |  |  |
| **Polypteriformes** |  |  |  |
| Polypteridae | *Calamoichthys calabaricus*  valid as *Erpetoichthys calabaricus* Smith, 1865 | absent | [59] |
|  | *Polypterus senegalis*  valid as *Polypterus senegalus* Cuvier, 1829 | absent | [59] |
| **Acipenseriformes** |  |  |  |
| Acipenseridae | *Acipenser baerii* Brandt, 1869 | absent | [60] |
|  | *Acipenser rubicundus*  valid as *Acipenser fulvescens* Rafinesque, 1817 | absent | [8] |
|  | *Acipenser Sturio*  valid as *Acipenser oxyrinchus* Mitchill, 1815 | absent | [10] |
|  | *Scaphirhynchus platyrhynchus*  valid as *Scaphirhynchus platorynchus* (Rafinesque, 1820) | absent | [3] |
| Polyodontidae | *Polyodon spathula* (Walbaum, 1792) | absent | [3] |
| **Holostei** |  |  |  |
| **Lepisosteiformes** |  |  |  |
| Lepisosteidae | *Lepisosteus osseus* (Linnaeus, 1758) | absent | [3] |
|  | *Lepisosteus platostomus* Rafinesque, 1820 | absent | [3] |
| **Amiiformes** |  |  |  |
| Amiidae | *Amia calva* Linnaeus, 1766 | absent | [1,8] |
| **Teleostei** |  |  |  |
| **Elopomorpha** |  |  |  |
| **Anguilliformes** |  |  |  |
| Anguillidae | *Anguilla anguilla* (Linnaeus, 1758) | absent | [3,10,25,61] |
|  | *Anguilla chrysypas*  valid as *Anguilla rostrata* (Lesueur, 1817) | absent | [8,47] |
| Congridae | *Conger japonicus*  valid as *Conger myriaster* (Brevoort, 1856) | absent | [47] |
|  | *Muraena conger*  valid as *Conger conger* (Linnaeus, 1758) | absent | [10] |
| Elopidae | *Elops saurus* Linnaeus, 1766 | absent | [8,62] |
| Muraenidae | *Lycodontis funebris*  valid as *Gymnothorax funebris* Ranzani, 1839 | absent | [8] |
|  | *Muraena (Gymnothorax) tessellata*  valid as *Gymnothorax favagineus* Bloch & Schneider, 1801 | ? | [63] |
|  | *Muraena (Gymnothorax) macrura*  valid as *Strophidon sathete* (Hamilton, 1822) | ? | [63] |
| Eurypharyngidae | *Gastrostomus bairdi*  valid as *Eurypharynx pelecanoides* Vaillant, 1882 | absent | [64] |
| **Osteoglossomorpha** |  |  |  |
| **Hiodontiformes** |  |  |  |
| Hiodontidae | *Amphiodon alosoides*  valid as *Hiodon alosoides* (Rafinesque, 1819) | absent | [3,65] |
|  | *Hiodon tergisus* Lesueur, 1818 | absent | [8] |
| **Osteoglossiformes** |  |  |  |
| Mormyridae | *Gnathonemus petersii* (Günther, 1862) | absent | [3] |
| Notopteridae | *Chitala chitala* (Hamilton, 1822) | absent | [66-68] |
|  | *Notopterus notopterus* (Pallas, 1769) | absent | [49] |
|  | *Notopterus afer*  valid as *Papyrocranus afer* (Günther, 1868) | absent | [3] |
| Osteoglossidae | *Osteoglossum bicirrhosum* (Cuvier, 1829) | absent | [3] |
|  | *Osteoglossum ferreirai* Kanazawa, 1966 | absent | [3] |
|  | *Scleropages jardinii* (Saville-Kent, 1892) | absent | [68] |
| Pantodontidae | *Pantodon buchholzi* Peters, 1876 | absent | [68] |
| **Alepocephaliformes** |  |  |  |
| Alepocephalidae | *Alepocephalus bicolor*Alcock, 1891 | absent | present work |
| **Clupeiformes** |  |  |  |
| Clupeidae | *Alausa vulgaris*  valid as *Alosa alosa* (Linnaeus, 1758) | absent | [10] |
|  | *Clupea harengus* Linnaeus, 1758 | absent | [3,8,10] |
| Engraulidae | *Engraulis anchoita* Hubbs & Marini, 1935 | absent | [69] |
| **Ostariophysii** |  |  |  |
| **Gonorynchiformes** |  |  |  |
| **Chanidae** | *Chanos chanos* (Fabricius 1775) | absent* | present work |
| **Cypriniformes** |  |  |  |
| Catostomidae | *Catostomus commersonii* (Lacepède, 1803) | present | [8,65] |
|  | *Moxostoma macrolepidotum* (Lesueur, 1817) | present | [8] |
| Cobitidae | *Cobitis fossilis*  valid as *Misgurnus fossilis* (Linnaeus, 1758) | absent | [10] |
| Cyprinidae | *Barbus conchonius*  valid as *Pethia conchonius*(Hamilton, 1822) | present | [70] |
|  | *Barbus fluviatilis*  *valid as Barbus barbus*(Linnaeus, 1758) | absent | [10] |
|  | *Carassius carassius* (Linnaeus, 1758) | absent | [43] |
|  | *Carassius carassius longsdorfi* (Linnaeus, 1758) | present | [72] |
|  | *Cyprinus auratus*  valid as *Carassius auratus* (Linnaeus, 1758) | absent | [10] |
|  | *Carassius auratus* (Linnaeus, 1758) | present | [71] |
|  | *Cyprinus carpio* Linnaeus, 1758 | absent | [8,10,12,43] |
|  | *Cyprinus carpio* Linnaeus, 1758 | present | [71,74] |
|  | *Labeo bata* (Hamilton, 1822) | absent | [67] |
|  | *Labeo rohita* (Hamilton, 1822) | absent | [49] |
| Leuciscidae | *Chondrostoma nasus*  valid as *Chondrostoma angorense* Elvira, 1987 | present | [10] |
|  | *Cyprinus brama*  valid as *Abramis brama* (Linnaeus, 1758) | present | [16] |
|  | *Abramis Brama*  valid as *Abramis brama* (Linnaeus, 1758) | present | [10] |
|  | *Leuciscus rutilus*  valid as *Rutilus rutilus* (Linnaeus, 1758) | present | [10] |
|  | *Notemigonus crysoleucas* (Mitchill, 1814) | absent | [75] |
|  | *Phoxinus laevis*  valid as *Phoxinus phoxinus* (Linnaeus, 1758) | absent | [10] |
|  | *Squalius cephalus* (Linnaeus, 1758) | present | [10] |
| Nemacheilidae | *Cobitis barbatula*  valid as *Barbatula barbatula* (Linnaeus, 1758) | present | [10] |
| Xenocyprididae | *Ctenopharyngodon idella* (Valenciennes, 1844) | absent | [41] |
| Danionidae | *Danio rerio* (Hamilton, 1822) | present | [76] |
| Gobionidae | *Gobio fluviatilis*  valid as *Gobio gobio* (Linnaeus, 1758) | present | [10] |
| Tincidae | *Tinca tinca* (Linnaeus, 1758) | absent | [42] |
|  | *Tinca vulgaris*  valid as *Tinca tinca* (Linnaeus, 1758) | present | [10] |
| **Characiformes** |  |  |  |
| Serrasalmidae | *Colossoma macropomum* (Cuvier, 1816) | present | [55] |
|  | *Piaractus mesopotamicus* (Holmberg, 1887) | present | [55] |
| **Siluriformes** |  |  |  |
| Bagridae | *Mystus seenghala* (Sykes, 1839)  valid as *Sperata seenghala* (Sykes, 1839) | absent | [77] |
|  | *Mystus vittatus* (Bloch, 1794) | absent | [49] |
|  | *Sperata aor* (Hamilton, 1822) | absent | [67] |
| Clariidae | *Clarias batrachus*  valid as *Clarias magur* (Hamilton, 1822) | absent | [78] |
|  | *Clarias gariepinus* (Burchell, 1822) | absent | [50] |
| Heptapteridae | *Rhamdia quelen* (Quoy & Gaimard, 1824) | absent | [51] |
| Ictaluridae | *Ictalurus punctatus* (Rafinesque, 1818) | present | [48,79-80] |
|  | *Ictalurus punctata*  valid as *Ictalurus punctatus* (Rafinesque, 1818) | present | [81] |
|  | *Ameiurus lacustris*  valid as *Ameiurus nebulosus* (Lesueur, 1819) | present | [8] |
|  | *Amiurus nigricans*  valid as *Ameiurus nebulosus* (Lesueur, 1819) | present | [82] |
|  | *Ictalurus nebulosus* (Lesueur, 1819)  valid as *Ameiurus nebulosus* (Lesueur, 1819) | present | [48,83] |
| Pimelodidae | *Hemisorubim platyrhynchos* (Valenciennes, 1840) | present | [46] |
| Siluridae | *Parasilurus asotus*  valid as *Silurus asotus* Linnaeus, 1758 | absent | [47] |
|  | *Silurus asotus* Linnaeus, 1758 | present | [84] |
|  | *Silurus glanis* Linnaeus, 1758 | absent | [43] |
| Trichomycteridae | *Trichomycterus brasiliensis* Lütken, 1874 | present | [45] |
| **Esociformes** |  |  |  |
| Esocidae | *Esox americanus* Gmelin, 1789 | absent | [85] |
|  | *Esox lucius* Linnaeus, 1758 | absent | [10,86] |
|  | *Lucius masquinongy*  valid as *Esox masquinongy* Mitchill, 1824 | absent | [8,85] |
|  | *Esox niger* Lesueur, 1818 | absent | [85] |
| Umbridae | *Umbra limi* (Kirtland, 1840) | absent | [85] |
| **Salmoniformes** |  |  |  |
| Salmonidae | *Oncorhynchus gorbuscha* (Walbaum, 1792) | present | [43] |
|  | *Oncorhynchus mykiss* (Walbaum, 1792) | present | [71,87] |
|  | *Coregonus oxyrhynchus* (Linnaeus, 1758) | absent | [10] |
|  | *Salmo gairdneri*  valid as *Oncorhynchus mykiss* (Walbaum, 1792) | present | [3,88] |
|  | *Trutta Fario*  valid as *Salmo trutta* Linnaeus, 1758 | present | [10] |
|  | *Salmo salar*  valid as *Salmo trutta* Linnaeus, 1758 | absent | [8] |
|  | *Salmo trutta* Linnaeus, 1758 | present | [3,71] |
|  | *Salvelinus fontinalis* (Mitchill, 1814) | present | [3] |
|  | *Cristivomer namaycush*  valid as *Salvelinus namaycush* (Walbaum, 1792) | absent | [8] |
| **Osmeriformes** |  |  |  |
| Osmeridae | *Osmerus Eperlanus*  valid as *Osmerus eperlanus* (Linnaeus, 1758) | ? | [10] |
| Plecoglossidae | *Plecoglossus altivelis* (Temminck & Schlegel, 1846) | absent | present work |
| **Galaxiiformes** |  |  |  |
| Galaxiidae | *Galaxias maculatus*(Jenyns, 1842) | absent | present work |
| **Argentiniformes** |  |  |  |
| Argentinidae | *Argentina kagoshimae* Jordan & Snyder, 1902 | absent | present work |
|  | *Argentina sialis* Gilbert, 1890 | absent | [89] |
|  | *Argentina sphyraena* Linnaeus, 1758 | absent | [89] |
| **Stomiformes** |  |  |  |
| Gonostomatidae | *Gonostoma bathyphilum, Gonostoma grande*  both valid as *Sigmops bathyphilus* (Vaillant ,1884) | absent | [64] |
|  | *Gonostoma elongatum*  valid as *Sigmops elongatus* (Günther, 1878) | absent | [64] |
| Stomiidae | *Chauliodus Sloanei*  valid as *Chauliodus schmidti* Ege, 1948 | absent | [64] |
|  | *Malacosteus niger* Ayres, 1848 | absent | [64] |
|  | *Stomias boa* (Risso, 1810) | absent | [64] |
| **Ateleopodiformes** |  |  |  |
| Ateleopodidae | *Ateleopus japonicus* Bleeker, 1853 | present | present work |
|  | [*Ateleopus*](http://researcharchive.calacademy.org/research/ichthyology/catalog/fishcatget.asp?genid=1931) [*purpureus*](http://researcharchive.calacademy.org/research/ichthyology/catalog/fishcatget.asp?spid=28535) [Tanaka](http://en.wikipedia.org/wiki/Shigeho_Tanaka), [1915](http://researcharchive.calacademy.org/research/ichthyology/catalog/getref.asp?id=4324) | present | present work |
| **Aulopiformes** |  |  |  |
| Giganturidae | *Bathypterus lisae*  valid as *Gigantura indica* Brauer, 1901 | absent | [90] |
|  | *Gigantura vorax*  valid as *Gigantura chuni* Brauer, 1901 |  | [90] |
| Synodontidae | *Saurida elongata* (Temminck & Schlegel, 1846) | absent | present work |
|  | *Saurida micropectoralis* Shindo & Yamada, 1972 | absent | present work |
|  | *Saurida tumbil* (Bloch, 1795) | absent | [91] |
| **Myctophiformes** |  |  |  |
| Myctophidae | *Gymnoscopelus braueri* (Lönnberg, 1905) | present | present work |
|  | *Stenobrachius leucopsarus* (Eigenmann & Eigenmann, 1890) | present | [92] |
| **Acanthomorpha** |  |  |  |
| **Lampriformes** |  |  |  |
| Trachipteridae | *Trachipterus ishikawae* Jordan & Snyder, 1901 | present | [93] |
| Lophotidae | *Eumecichthys fiskii*  valid as *Eumecichthys fiski* (Günther, 1890) | present | [94] |
| **Gadiformes** |  |  |  |
| Gadidae | *Gadus Merlangus*  valid as *Merlangius merlangus* (Linnaeus, 1758) | present | [11] |
|  | *Gadus morhua* Linnaeus, 1758  (with *Gadus callarias* being a synonym of *Gadus morhua*) | present | [8,10,12,16,71,87,95-98] |
|  | *Gadus pollachius*  *= Gadus Carbonarius,* L.  valid as *Pollachius virens.* (Linnaeus, 1758) | present | [10,97] |
|  | *Pollachius virens.* (Linnaeus, 1758) | present | [8,12,96], |
|  | *Melanogrammus aeglefinus* (Linnaeus, 1758) | present | [8,12] |
|  | *Microgradus tomcod* (Walbaum, 1792) | present | [8] |
| Lotidae | *Brosme brosme* (Ascanius, 1772) | present | [97] |
|  | *Gadus lota*  valid as *Lota lota* (Linnaeus, 1758) | present | [10] |
|  | *Molva bjerkelange*  valid as *Molva molva* (Linnaeus, 1758) | present | [97] |
|  | *Motella tricirrata* (non Brünnich, 1768)  misapplied name of *Gaidropsarus vulgaris* (Cloquet, 1824) | present | [9] |
| Bathygadidae | *Bathygadus antrodes* (Jordan & Starks, 1904) | present | present work |
| Merlucciidae | *Merluccius bilinearis*  valid as *Merluccius albidus* (Mitchill, 1818) | present | [8] |
|  | *Gadus merluchius*  valid as *Merluccius merluccius* (Linnaeus, 1758) | present | [10] |
| Gaidropsaridae | *Onos mustela*  valid as *Ciliata mustela* (Linnaeus, 1758) | present | [12] |
| **Zeiformes** |  |  |  |
| Zeidae | *Zeus faber* Linnaeus, 1758 | present | [7,10,12] |
| **Trachichthyiformes** |  |  |  |
| Diretmidae | *Diretmoides veriginae* Kotlyar, 1987 | present | present work |
| **Beryciformes** |  |  |  |
| Berycidae | *Beryx mollis* Abe, 1959 | present | present work |
| Holocentridae | *Holocentrus ascensionis*  valid as *Holocentrus adscensionis* (Osbeck, 1765) | present | [8] |
| **Ophidiiformes** |  |  |  |
| Carapidae | *Carapus acus* (Brünnich, 1768) | present | [99] |
| Ophidiidae | *Ophidion barbatum* Linnaeus, 1758) | present | present work |
| **Batrachoidiformes** |  |  |  |
| Batrachoididae | *Halobatrachus didactylus* (Bloch & Schneider, 1801) | present | [100] |
|  | *Opsanus tau* (Linnaeus, 1766) | present | [8,101] |
| **Gobiiformes** |  |  |  |
| Gobiidae | *Gillichthys mirabilis* Cooper, 1864 | present | [102-103] |
|  | *Gobius niger* Linnaeus, 1758 | ? | [10] |
| **Syngnathiformes** |  |  |  |
| Callionymidae | *Callionymus lyra* Linnaeus, 1758 | present | [12] |
| Dactylopteridae | *Dactyloptera volitans*  valid as *Dactylopterus volitans* (Linnaeus, 1758) | ? | [10] |
| Mullidae | *Upeneus martinicus*  valid as *Mulloidichthys martinicus* (Cuvier, 1829) | present | [8] |
|  | *Mullus surmuletus* Linnaeus, 1758 | present | [10] |
| Syngnathidae | *Nerophis aequoreus*  invalid name, species unidentified | present | [7,8,12,96] |
|  | *Syngnathus acus* Linnaeus, 1758 | present | [7,8,12,96] |
|  | *Syphonostoma typhle*  valid as *Syngnathus typhle* (Linnaeus, 1758) | present | [7,8,12,96] |
| **Scombriformes** |  |  |  |
| Gempylidae | *Promethichthys prometheus* (Cuvier, 1832) | present | present work |
| Scombridae | *Katsuwonus pelamis* (Linnaeus, 1758) | present | [22,104] |
|  | *Gymnosarda alleterata*  valid as *Euthynnus alletteratus* (Rafinesque, 1810) | present | [8] |
|  | *Scomber scombrus* Linnaeus, 1758 | present | [8,10,96], |
|  | *Scomber japonicus* Houttuyn, 1782 | present | [105] |
|  | *Scomber tapeinocephalus*  valid as *Scomber australasicus* Cuvier, 1832 | present | [105] |
|  | *Scomberomorus maculatus* (Mitchill, 1815) | present | [8] |
|  | *Scomberomorus cavalla* (Cuvier, 1829) | present | [8] |
|  | *Thunnus obesus* (Lowe, 1839) | present | [106] |
|  | *Thunnus thynnus* (Linnaeus, 1758) | present | [107-108] |
| Trichiuridae | *Trichiurus japonicus* Temminck & Schlegel, 1844 | present | present work |
| **Synbranchiformes** |  |  |  |
| Channidae | *Channa punctatus*  valid as *Channa punctata* (Bloch, 1793) | present | [109] |
| Osphronemidae | *Osphronemus gorami*  valid as *Osphronemus goramy* Lacepède, 1801 | present | [110] |
| **Carangiformes** |  |  |  |
| Menidae | *Mene maculata* (Bloch & Schneider, 1801) | present | present work |
| Sphyraenidae | *Sphyraena barracuda* (Edwards, 1771) | present | [8,96] |
| **Carangoidea** |  |  |  |
| Carangidae | *Caranx hippos* (Linnaeus, 1766) | present | [8] |
|  | *Scomber trachurus*  valid as *Trachurus trachurus* (Linnaeus, 1758) | present | [10] |
|  | *Selene vomer* (Linnaeus, 1758) | present | [8] |
|  | *Seriola quinqueradiata* Temminck & Schlegel, 1845 | present | [111] |
| Coryphaenidae | *Coryphaena hippurus* Linnaeus, 1758 | present | [112] |
| Echeneidae | *Echeneis naucrates* Linnaeus, 1758 | present | [113] |
| Rachycentridae | *Rachycentron canadum* (Linnaeus, 1766) | present | [114] |
| **Xiphioidea** |  |  |  |
| Istiophoridae | *Istiophorus platypterus* (Shaw, 1792) | present | [115] |
| **Pleuronectoidea** |  |  |  |
| Paralichthyidae | *Paralichthys olivaceus* (Temminck & Schlegel, 1846) | present | [116] |
| Pleuronectidae | *Hippoglossus hippoglossus* (Linnaeus, 1758) | present | [7,8,12,96], |
|  | *Depranopsetta platessoides*  valid as *Hippoglossoides platessoides* (Fabricius, 1780) | present | [97] |
|  | *Kareius bicoloratus*  valid as *Platichthys bicoloratus* (Basilewsky, 1855) | present | [117] |
|  | *Limanda limanda* (Linnaeus, 1758) | present | [118] |
|  | *Limanda yokohamae*  valid as *Pseudopleuronectes yokohamae* (Günther, 1877) | present | [119] |
|  | *Limmanda herzensteini*  valid as *Pseudopleuronectes herzensteini* (Jordan & Snyder, 1901) | present | [117] |
|  | *Pleuronectes cynoglossus*  valid as *Glyptocephalus cynoglossus* (Linnaeus, 1758) | present | [97] |
|  | *Platichthys flesus* (Linnaeus, 1758) | present | [120] |
|  | *Pleuronectus flesus*  valid as *Pleuronectes platessa* Linnaeus, 1758 | present | [121] |
|  | *Pleuronectes Flesus*  valid as *Pleuronectes platessa* Linnaeus, 1758 | present | [10,97] |
|  | *Pleuronectes platessa* Linnaeus, 1758 | present | [7,10,12,16,96] |
|  | *Pleuronectus platessa*  valid as *Pleuronectes platessa* Linnaeus, 1758 | present | [121] |
|  | *Pseudopleuronectes americanus* (Walbaum, 1792) | present | [8] |
|  | *Verasper moseri* Jordan & Gilbert, 1898) | present | [117,122] |
| Scophthalmidae | *Pleuronectes maximus*  valid as *Scophthalmus maximus* (Linnaeus, 1758) | present | [10,16] |
|  | *Scophthalmus maximus* (Linnaeus, 1758) | present | [123] |
|  | *Rhombus laevis*  valid as *Scophthalmus rhombus* (Linnaeus, 1758) | present | [9] |
|  | *Pleuronectes Rhombus*  valid as *Scophthalmus rhombus* (Linnaeus, 1758) | present | [10,18] |
| Soleidae | *Pleuronectes solea*  valid as *Solea solea* (Linnaeus, 1758) | present | [10,18] |
|  | *Solea senegalensis* Kaup, 1858 | present | [124-125] |
| **Blenniiformes** |  |  |  |
| **Blennioidei** |  |  |  |
| Blenniidae | *Blennius gattorugine*  valid as *Parablennius gattorugine* (Linnaeus, 1758) | present | [133-134] |
| **Cichloidei** |  |  |  |
| Pomacentridae | *Abudefduf saxatilis* (Linnaeus, 1758) | present | [8] |
| Cichlidae | *Haplochromis sp.*  valid as *Allochromis* Greenwood (1980) | present | [126] |
|  | *Oreochromis niloticus* (Linnaeus, 1758) | present | [14] |
|  | *Pelvicachromis pulcher* (Boulenger, 1901) | present | [126] |
| Mugilidae | *Mugil auratus*  valid as *Chelon auratus* (Risso, 1810) | present | [132] |
|  | *Mugil cephalus* Linnaeus, 1758 | present | [8] |
|  | *Mugil saliens*  valid as *Chelon saliens* (Risso, 1810) | present | [132] |
| **Atherinoidea** |  |  |  |
| Atherinidae | *Atherina presbyter* Cuvier, 1829 | present | [10] |
| **Belonoidea** |  |  |  |
| Adrianichthyidae | *Oryzias latipes* (Temminck & Schlegel, 1846) | present | [127] |
| Belonidae | *Belone longirostris*  valid as *Belone belone* (Linnaeus, 1760). | present | [10,16] |
|  | *Tylosaurus marinus*  valid as *Strongylura marina* (Walbaum, 1792) | absent | [8] |
| Exocoetidae | *Cypselurus agoo*  valid as *Cheilopogon agoo* (Temminck & Schlegel 1846) | absent | [128] |
| **Cyprinodontoidea** |  |  |  |
| Fundulidae | *Fundulus heteroclitus* (Linnaeus, 1766) | present | [3] |
|  | *Fundulus heteroclitus* (Linnaeus, 1766) | absent | [85] |
|  | *Fundulus majalis* (Walbaum, 1792) | absent | [85] |
| Poeciliidae | *Gambusia affinis* (Baird & Girard, 1853) | present | [128] |
|  | *Xiphophorus helleri*  valid as *Xiphophorus alvarezi* Rosen, 1960 | present | [3,130-131] |
| **Acanthuriformes** |  |  |  |
| **Acanthuroidei** |  |  |  |
| Lutjanidae | *Ocyurus chrysurus*  valid as *Lutjanus ambiguus* (Poey, 1860) | present | [8] |
|  | *Neomanis apodus*  valid as *Lutjanus apodus* (Walbaum, 1792) | present | [8] |
|  | *Neomanis griseus*  valid as *Lutjanus griseus* (Linnaeus, 1758) | present | [8,135] |
| Sciaenidae | *Aplodinotus grunniens* Rafinesque, 1819 | present | [8] |
|  | *Chrysochir aurea* (Richardson, 1846) | present | present work |
|  | *Menticirrhus americanus* (Linnaeus, 1758) | present | [8] |
| Chaetodontidae | *Chaetodon ocellatus* Bloch, 1787 | present | [8] |
| Pomacanthidae | *Angelichthys isabelita*  valid as *Holacanthus bermudensis* Goode, 1876 | present | [8] |
|  | *Pomacanthus arcuatus* (Linnaeus, 1758) | present | [8] |
|  | *Pomacanthus paru* (Bloch, 1787) | present | [8] |
| Ephippidae | *Chaetodipterus faber* (Broussonet, 1782) | present | [8] |
| Acanthuridae | *Teuthys caeruleus*  valid as *Acanthurus caeuleus* Bloch & Schneider, 1801 | present | [8] |
| Haemulidae | *Anisotremus virginicus* (Linnaeus, 1758) | present | [8] |
|  | *Haemulon album* Cuvier, 1830 | present | [8] |
|  | *Haemulon parra* (Desmarest, 1823) | present | [8] |
|  | *Haemulon plumieri* (Lacepède, 1801) | present | [8] |
|  | *Haemulon sciurus* (Shaw, 1803) | present | [8] |
| Malacanthidae | *Malacanthus plumieri* (Bloch, 1786) | present | [8] |
| Moronidae | *Labrax Lupus*  valid as *Dicentrarchus labrax* (Linnaeus, 1758) | present | [10] |
|  | *Dicentrarchus labrax* (Linnaeus, 1758) | present | [137-138] |
|  | *Roccus chrysops*  valid as *Morone chrysops* (Rafinesque, 1820) | present | [8] |
|  | *Morone americana* (Gmelin, 1789) | present | [8] |
|  | *Morone saxatilis* (Walbaum, 1792) | present | [139] |
| Sparidae | *Maena sp*  valid as *Spicara maena* (Linnaeus, 1758) | present | [10] |
|  | *Calamus bajonado* (Bloch & Schneider, 1801) | present | [8] |
|  | *Chrysophris Aurata*  valid as *Sparus aurata* Linnaeus, 1758 | present | [10] |
|  | *Lagodon rhomboides* (Linnaeus, 1766) | present | [8] |
|  | *Sparus aurata* Linnaeus, 1758 | present | [141-142] |
|  | *Sparus erythrinus*  valid as *Pagellus erythrinus* (Linnaeus, 1758) | present | [10] |
| **Lophoidei** |  |  |  |
| Lophiidae | *Lophius americanus* Valenciennes, 1837 | present | [8,96,143] |
|  | *Lophius piscatorius* Linnaeus, 1758 | present | [7,8,12]  present work |
|  | *Lophiomus setigerus* (Vahl, 1797) | present | present work |
| **Tetraodontoidei** |  |  |  |
| Balistiidae | *Balistes carolinensis*  valid as *Balistes capriscus* Gmelin, 1789 | present | [8] |
|  | *Balistes vetula* Linnaeus, 1758 | present | [8] |
|  | *Canthidermis rotundatus* (Marion de Procé, 1822) | present | [144] |
|  | *Melichthys niger* (Bloch, 1786) | present | [8] |
| Diodontidae | *Diodon hystrix* Linnaeus, 1758 | present | [8] |
|  | *Diodon holacanthus* Linnaeus, 1758 | present | [145] |
| Molidae | *Orthagoriscus molae*  valid as *Mola mola* (Linnaeus, 1758) | present | [9] |
| Monacanthidae | *Monacanthus ciliatus* (Mitchill, 1818) | present | [8] |
|  | *Monacanthus hispidus*  valid as *Stephanolepis hispidus* (Linnaeus, 1766) | present | [8] |
| Ostraciidae | *Lactophrys tricornis* valid as *Acanthostracion quadricornis* (Linnaeus, 1758) | present | [8] |
| Tetraodontidae | *Takifugu niphobles*  valid as *Gastrophysus niphobles* (Jordan & Snyder, 1901) | present | [145] |
|  | *Spheroides spengleri*  valid as *Sphoeroides spengleri* (Bloch, 1785) | present | [8] |
|  | *Fugu rubipres rubipres*  valid as *Takifugu rubripes* (Temminck & Schlegel, 1850) | present | [146] |
| **Centrarchiformes** |  |  |  |
| Centrarchidae | *Ambloplites rupestris* (Rafinesque, 1817) | present | [2,8,65] |
|  | *Micropterus dolomieu* Lacepède, 1802 | present | [8] |
|  | *Micropterus salmoides* (Lacepède 1802) | present | [136] |
| Sinipercidae | *Siniperca scherzeri* Steindachner, 1892 | present | [84] |
| Kyphosidae | *Kyphosus sectatrix*  valid as *Kyphosus bosquii* (Lacepède 1802) | present | [8] |
| **Labriformes** |  |  |  |
| Labridae | *Bodianus fulvus ruber*  valid as *Bodianus rufus* (Linnaeus, 1758) | present | [8] |
|  | *Choerodon azurio* (Jordan & Snyder, 1901) | absent | present work |
|  | *Choerodon schoenleinii* (Valenciennes, 1839) | absent | present work |
|  | *Cheilinus undulates*  valid as *Cheilinus undulatus* Rüppell, 1835 | absent | [57] |
|  | *Crenilabrus melops*  valid as *Symphodus melops* (Linnaeus, 1758) | present | [19] |
|  | *Iridio radiates*  valid as *Halichoeres radiatus* (Linnaeus (1758) | present | [8] |
|  | *Labrus Bailloni*  valid as *Symphodus bailloni* (Valenciennes, 1839) | present | [10] |
|  | *Labrus Vetula*  valid as *Labrus mixtus* Linnaeus, 1758 | present | [10] |
|  | *Tautogolabrus adspersus* (Walbaum, 1792) | absent | [56] |
| “Scarinae” | *Pseudoscarus guacamaia*  valid as *Scarus hoefleri* (Steindachner, 1881) | absent | [8,96] |
|  | *Sparisoma hoplomystax*  valid as *Sparisoma radians* (Valenciennes, 1840) | absent | [8,96] |
|  | *Scarus caerulus*  valid as *Scarus coeruleus* (Edwards, 1771) | absent | [8,96] |
|  | *Scarus ghobban* Forsskål, 1775 | absent | [57] present work |
|  | *Chlorurus sordidus* (Forsskål, 1775) | absent | [140] |
| Ammodytidae | *Ammodytes tobianus* Linnaeus, 1758 | present | [12] |
| **Perciformes** |  |  |  |
| Anarhichadidae | *Anarrichas latifrons*  valid as *Anarhichas denticulatus* Krøyer, 1845 | present | [97] |
|  | *Anarrhichas minor*  valid as *Anarhichas minor* Olafsen, 1772 | present | [97] |
|  | *Anarhichas lupus* Linnaeus, 1758 | present | [7,8,11,12] |
| Pholidae | *Pholis gunnellus* (Linnaeus, 1758) | present | [7,8,12] |
| Stichaeidae | *Chirolophis galerita*  valid as *Chirolophis ascanii* (Walbaum, 1792) | present | [8,12] |
| Zoarcidae | *Zoarces viviparus* (Linnaeus, 1758) | present | [7,8,12] |
|  | *Zoarces americanus* (Bloch & Schneider, 1801) | present | [8] |
| Agonidae | *Agonus cataphractus* (Linnaeus, 1758) | present | [8,12] |
|  | *Hemitripterus americanus* (Gmelin, 1789) | present | [8] |
| Cottidae | *Cottus gobio* Linnaeus, 1758 | present | [148] |
|  | *Cottus quadricornis*  valid as *Myoxocephalus quadricornis* (Linnaeus, 1758) | present | [149] |
|  | *Cottus scorpius*  valid as *Myoxocephalus scorpius* (Linnaeus, 1758) | present | [10,12,16,120,150] |
|  | *Myoxocephalus scorpius* (Linnaeus 1758) | present | [8,151] |
|  | *Myoxocephalus bubalis*  valid as *Taurulus bubalis* (Euphrasen, 1786) | present | [82] |
|  | *Parenophrys bubalis*  valid as *Taurulus bubalis* (Euphrasen, 1786) | present | [148] |
|  | *Myoxocephalus octodecemspinosus* (Mitchill, 1814) | present | [8] |
| Cyclopteridae | *Cyclopterus lumpus* Linnaeus, 1758 | present | [7,8,10,12,97] |
| Hexagrammidae | *Ophiodon elongatus* Girard, 1854 | present | [152] |
| Liparidae | *Cyclogaster montagui*  valid as *Liparis montagui* (Donovan, 1804) | present | [8,12] |
| Scorpaenidae | *Scorpaena porcus* Linnaeus, 1758 | present | [153] |
|  | *Scorpaena scrofa* Linnaeus, 1758 | present | [153] |
|  | *Scorpaenopsis neglecta* Heckel, 1837 | present | present work |
| Sebastidae | *Sebastes marinus (norvegicus)*  valid as *Sebastes norvegicus* (Ascanius, 1772) | present | [97] |
| Triglidae | *Trigla Gurnardus*  valid as *Eutrigla gurnardus* (Linnaeus, 1758) | ? | [10] |
|  | *Trigla Hirundo*  valid as *Chelidonichthys lucerna* (Linnaeus, 1758) | present | [10] |
|  | *Trigla lyra* Linnaeus, 1758 | present | [10] |
| Serranidae | *Diplectrum formosum* (Linnaeus, 1766) | present | [8] |
|  | *Epinephelus adscensionis* (Osbeck, 1765) | present | [8] |
|  | *Epinephelus chlorostigma* (Valenciennes, 1828) | present | [154] |
|  | *Epinephelus morio* (Valenciennes, 1828) | present | [8] |
|  | *Serranus cabrilla* (Linnaeus, 1758) | present | [155] |
| Trachinidae | *Trachinus draco* Linnaeus, 1758 | present | [10] |
| Gasterosteidae | *Gasterosteus laevis*  valid as *Pungitius laevis* (Cuvier, 1829) | present | [10] |
| Percidae | *Perca flavescens* (Mitchill, 1814) | present | [8] |
|  | *Perca fluviatilis* Linnaeus, 1758 | present | [10,13,16] |
|  | *Stizostedion canadense*  valid as *Sander canadensis* (Griffith & Smith, 1834) | present | [8] |
|  | *Stizostedion vitreum*  valid as *Sander vitreus* (Mitchill, 1818) | present | [8] |
| Channichtyidae | *Chionodraco hamatus* (Lönnberg, 1905) | present | [155] |
|  | *Cryodraco antarcticus* Dollo, 1900 | present | [155] |
|  | *Pagetopsis macropterus* (Boulenger, 1907) | present | [155] |
| Nototheniidae | *Dissostichus mawsoni* Norman, 1937 | present | [156-157] |
|  | *Notothenia angustata* Hutton, 1875 | present | [156] |
|  | *Notothenia coriiceps* Richardson, 1844 | present | [156] |
|  | *Pagothenia borchgrevinki* (Boulenger, 1902) | present | [156] |
|  | *Pleuragramma antarcticum* Boulenger, 1902 | present | [155-156] |
|  | *Trematomus bernacchii*  valid as *Pseudotrematomus bernacchii* (Boulenger, 1902) | present | [156] |
|  | *Trematomus hansoni*  valid as *Pseudotrematomus hansoni* (Boulenger 1902) | present | [156] |
|  | *Trematomus newnesi* Boulenger, 1902 | present | [155] |
|  | *Trematomus nicolai*  valid as *Pseudotrematomus nicolai* (Boulenger, 1902) | present | [156] |
|  | *Trematomus loennbergii*  valid as *Pseudotrematomus loennbergii* (Regan, 1913) | present | [156] |

**Supplementary reference list**

59. Conlon, J. M., Fan, H., & Fritzsch, B. Purification and structural characterization of insulin and glucagon from the Bichir *Polypterus senegalis* (Actinopterygii: Polypterifomes). *Gen. Comp. Endocrinol* **109**, 86-93 (1998). <https://doi.org/10.1006/gcen.1997.7007>

60. Daprà, F. et al. Siberian sturgeon (*Acipenser baeri*, Brandt JF 1869) gut: anatomic description. *Int. Aquat. Res.* **1**, 45-60 (2009).

61. Brinn, J. E. jr. The pancreatic islets of bony fishes. *Am. Zool*. **13**, 653-665 (1973). <https://doi.org/10.1093/icb/13.3.653>

62. Sekavec, G. B. Gross morphology of the digestive tract of the lady fish, *Elops saurus*. *Chesapeake Science* **12**, 275-276 (1971). <https://doi.org/10.2307/1350916>

63. Kenikar, V. A B D cells in extrapancreatic principal islets of Langerhans in two marine eels *Muraena tessellata* and *Muraena macrura. J. Biol. Sci*. **6**, 48-51 (1963).

64. Nusbaum-Hilarowicz, J. Études d’anatomie comparée sur les poissons provenant des campagnes scientifiques de S.A.S. le Prince de Monaco. Résultats des campagnes scientifiques accomplies sur son yacht par Albert Ier, prince souverain de Monaco 65, 1-100 (1923).

65. Al-Mahrouki, A. A. F. Characterization of the gastro-entero-pancreatic system of Osteoglossomorpha, an immunohistochemical, immunocytochemical, and molecular study. (University of Toronto, Canada, 2001).

66. Mitra, A., Mukhopadhyay, P. K., & Homechaudhuri, S. Histomorphological study of the gut developmental pattern in early life history stages of featherback, *Chitala chitala* (Hamilton). *Arch. Pol. Fish.* **23**, 25-35 (2015). https://doi.org/[10.1515/aopf-2015-0003](https://www.researchgate.net/deref/http%3A%2F%2Fdx.doi.org%2F10.1515%2Faopf-2015-0003?_sg%5B0%5D=oNMMWH-pdDcVtOGz_dIBo_y8mD11Q6GJHw2ORiq5tvLYYf6wwJRAXqz3OKgpkcGHNhw8AMq2M9W7-8AKefoGK67I9g.VMc-O0ru1hZRdIm3Qd3uF3noUFcB7U6GxNnRbcS9XVpAdgUUf4ZWaUIWdtoAJLdS-p2jMYoDWcrIRNDzc8H48A)

67. Gosh, S.K., Barun S. & Chakrabarti P. A comparative study of the histoarchitecture of endocrine pancreas in *Labeo bata* (Hamilton, 1822), *Sperata aor* (Hamilton, 1822) and *Chitala chitala* (Hamilton, 1822)*. Int. J. Aquat. Biol.* **4**, 17-24 (2016).

68. Al-Mahrouki, A. A., & Youson, J. H. Immunohistochemical studies of the endocrine cells within the gastro-entero-pancreatic system of Osteoglossomorpha, an ancient teleostean group. *Gen. Comp. Endocrinol*. **110**,125–139 (1998). https://doi.org/[10.1006/gcen.1998.7070](https://doi.org/10.1006/gcen.1998.7070)

69. Cohen, S., Diaz, M. V., & Díaz, A. O. Histological and histochemical study of the digestive system of the Argentine anchovy larvae (*Engraulis anchoita*) at different developmental stages of their ontogenetic development. *Acta Zool.* **95**, 409–420 (2014). <https://doi.org/10.1111/azo.12038>

70. Rombout, J. H. W. M., Rademakers L. H. P. M., & van Hees, J. P. Pancreatic endocrine cells of *Barbus conchonius* (Teleostei, Cyprinidae), and their relation to the enteroendocrine cells. *Cell Tissue Res.* **203**, 9-23 (1979). <https://doi.org/10.1007/BF00234325>

71. Jönsson, A.-C. Regulatory peptides in the pancreas of two species of elasmobranchs and in the Brockmann bodies of four teleost species. *Cell Tissue Res.* **266**, 163–172 (1991). <https://doi.org/10.1007/BF00678722>

72. Kobayashi, K., & Takahaski, Y. Light and electron microscope observations on the islets of Langerhans in *Carassius carussius longsdorfii*. *Arch. Histol. Jpn.* **31**, 433-454 (1970). <https://doi.org/10.1679/aohc1950.31.433>

73. Nakamura, M., & Yokote, M. Ultrastructural studies on the islets of Langerhans of the carp. *Z. Anat. Entwicklungsgesch.* **134**, 61-72 (1971). <https://doi.org/10.1007/BF00523287>

74. [Kong, H.-S](http://europepmc.org/search;jsessionid=FtQK3JRNUTpClwDibuxJ.1?page=1&query=AUTH:%22Kong+HS%22)., [Lee, J.-H](http://europepmc.org/search;jsessionid=FtQK3JRNUTpClwDibuxJ.1?page=1&query=AUTH:%22Lee+JH%22)., [Park K.-D](http://europepmc.org/search;jsessionid=FtQK3JRNUTpClwDibuxJ.1?page=1&query=AUTH:%22Park+KD%22)., [Ku, S.-K](http://europepmc.org/search;jsessionid=FtQK3JRNUTpClwDibuxJ.1?page=1&query=AUTH:%22Ku+SK%22)., & [Lee, H.-S](http://europepmc.org/search;jsessionid=FtQK3JRNUTpClwDibuxJ.1?page=1&query=AUTH:%22Lee+HS%22). Immunohistochemical study of the endocrine cells in the pancreas of the carp, *Cyprinus carpio* (Cyprinidae)*. J. Vet. Sci.* **3**, 303-314 (2002). https://doi.org/[10.4142/jvs.2002.3.4.303](https://www.researchgate.net/deref/http%3A%2F%2Fdx.doi.org%2F10.4142%2Fjvs.2002.3.4.303?_sg%5B0%5D=2z6il4d9ZGXBkjvs4KmuwUBwyhBqnvLK_HUZih-BX7ecZG7L4thT4tQkGgmcq2GrmnnaqIjmDFZ8byuaJozo-IGvuQ.iXYfoxQB-0gtHx9mgY94y-xE1_-LuDCBLyE-oelUzz43eV-DPMUZmlTWjRSm9F0O7WB6nc6qyea2_Za3isXS_w)

75. Yue, M. Y. Anatomical and histological studies on selected organs of golden shiner *Notemigonus Crysoleucas* (Mitchill). (Oklahoma State University, Stillwater, 1973).

76. Menke, A. L., Spitsbergen, J. M., Wolterbeek, A. P., & Woutersen, R. A. Normal anatomy and histology of the adult zebrafish. *Toxicol. Pathol*. **39**, 759-775 (2011). <https://doi.org/10.1177/0192623311409597>

77. Khanna, S. S., & Gill T. S. Effect of Glucose loading on the blood glucose level and histology of the principal islets in *Channa punctatus*. *Endocrinol. Japon.* **20**, 375-384 (1973).

78. Khanna, S. S., & Mehrotra, B. K. Effect of insulin and glucose on the beta cells of the pancreatic islets in fresh water teleost, *Clarias batrachus. Acta Zool.* **50**, 91-95 (1969). https://doi.org/[10.1111/j.1463-6395.1969.tb00532.x](https://www.researchgate.net/deref/http%3A%2F%2Fdx.doi.org%2F10.1111%2Fj.1463-6395.1969.tb00532.x?_sg%5B0%5D=iwKD_YhDnPlH1rwcYCbkyrz9K8gq3BVNoCgK66ca9G7TDp_dAdS0UWbmMWDwKMv48Nsn3_-JHhHOfKzofBUjHiPr-w.RfqG38clnPQF1Wgt4UNnaghiXRmDEt0S7bUkW5FOhqt7VzTPKg_BTzFs1mwMLXI8v3xHhnK9idph3kIUxpbzKQ)

79. Gammon, R. L. The gross and microanatomy of the digestive tract and pancreas of the channel catfish, *Ictalurus punctatus*. (Kansas State University, Manhattan, 1970).

80. Peterson, B. C., Bilodeau-Bourgeois, A. L., & Small, B. C. Response of the somatotropic axis to alterations in feed intake of channel catfish (*Ictalurus punctatus*). *Comp. Biochem. Physiol. A Mol. Integr. Physiol.* **153**, 457–463 (2009). [https://doi.org/10.1016/j.cbpa.2009.04.614](https://doi.org/10.1016/j.cbpa.2009.04.614)

81. Andrews, P.C. & Ronner P. Isolation and structures of glucagon and glucagon-like peptide from catfish pancreas. *J. Biol. Chem.* **260**, 3910-3914 (1985).

82. Vincent, S., Dodds, E. C., & Dickens, F. The pancreas of teleostean fishes and the source of insulin. *Q. J. Exp. Physiol.* **15**, 313–317 (1925). <https://doi.org/10.1113/expphysiol.1925.sp000360>

83. Le Discorde, D. Étude cytologique du pancréas endocrine de la barbotte brune (Pisces *Ictalurus nebulosus*, le Sueur 1819). *Anat. Histol. Embryol.* **6**, 150–156 (1977). <https://doi.org/10.1111/j.1439-0264.1977.tb00429.x>

84. Lee, J. H., Ku, S.K., & Lee, H. S. Comparative study of endocrine cells in the principal pancreatic islets of two teleosts, *Silurus asotus* (Siluridae) and *Siniperca scherzeri* (Centropomidae). *J. Vet. Sci.* **2**, 75–80 (2001).

85. Boldyreff, E. B. A microscopic study of the pancreas in fishes; especially those of the orders Haplomi and Cyprinodontes. *Copeia* **1935**, 23-34 (1935). <https://doi.org/10.2307/1436633>

86. Vorstman, A. The development of the pancreas, the gall bladder, the ductus choledochus and the air bladder of *Esox lucius* L. *Biol. Jaarb. Dodonaea* **15**, 87-105 (1948).

87. Jönsson, A.-C. Co-localization of peptides in the Brockmann bodies of the cod (*Gadus morhua*) and the rainbow trout (*Oncorhynchus mykiss*). *Cell Tissue Res.* **273**, 547–555 (1993). <https://doi.org/10.1007/BF00333708>

88. Librán-Pérez, M., López-Patiño, M. A., Míguez, J. M., & Soengas, J. L. Oleic acid and octanoic acid sensing capacity in rainbow trout *Oncorhynchus mykiss* is direct in hypothalamus and Brockmann bodies. *PLoS ONE,* 8(3):e59507 (2013). https://doi.org/[10.1371/journal.pone.0059507](https://doi.org/10.1371/journal.pone.0059507)

89. Cohen, D. M. A Revision of the fishes of the subfamily Argentininae. *Bull. Fla. State Mus.*, *Biol. Sci*. **3**, 93-172 (1958).

90. Walters, V. A contribution to the biology of the Giganturidae, with description of a new genus and species. *Bull. Mus. Comp. Zool.* **125**, 297–319 (1961).

91. Shyamasundari, K., Raja Kumari, V. J. V., & Hanumantha Rao, K. Observations on the pancreas of the marine lizard fish *Saurida tumbil* (Bloch). *J. Fish Biol*. **21**, 449-454 (1982). <https://doi.org/10.1111/j.1095-8649.1982.tb02851.x>

92. Jollie, M. T. The general anatomy of *Lampanyctus leucopsarus* (Eigenmann and Eigenmann). (Stanford University, Stanford, 1954).

93. Honma Y., Ushiki, T., Takeda, M., & Kubota, S. Histological studies on some organs of two male dealfishes, *Trachipterus ishikawae*, caught on the beach of Shirahama, Wakayama Prefecture, Pacific Coast of Japan. *Publ. Seto Mar. Biol. Lab.* **40**, 199-205 (2005). https://doi.org/[10.5134/176320](https://doi.org/10.5134/176320)

94. Honma, Y., Ushiki, T., & Takeda, T. Histology of the ink tube and its associated organs in a unicornfish, *Eumecichthys fiskii* (Lampridiformes). *Ichthyol. Res.* **40**, 19-25 (1999). <https://doi.org/10.1007/BF02674944>

95. Morrison, C. M. *Histology of the Atlantic Cod,* Gadus morhua*: An Atlas – Part 4: Eleutheroembryo and Larva.* (Canada NRC Publication, Ottawa, 1993).

96. McCormick, N. A. Insulin from fish. *Bulletin of the* *Biological Board of Canada* **7**, 3-23 (1924).

97. Caesar, R. Zur Zytologie der Inselorgane von Teleostiern, mit besonderer Berücksichtigung des Kolloidvorkommens. *Z. Zellforsch. Mikrosk. Anat.* **40**, 571–584 (1954).

98. Thomas, N. W. Morphology of endocrine cells in the islet tissue of the cod *Gadus callarias*. *Acta Endocrinol.* **63**,679-695 (1970). https://doi.org/[10.1530/acta.0.0630679](https://doi.org/10.1530/acta.0.0630679)

99. Emery, C. Fierasfer. Studî interno alla sistematica, l’anatomia e la biologia delle specie mediterranee di questo genere. *Memorie della Classe di scienze fisiche, matematiche e naturali* **7**, 167-254 (1880).

100. Palazón-Fernández, J. L., Suso, M. P., Mancera, J. M., Sarasquete, C. Immunohistochemical study of the principal pancreatic islet of the toadfish, *Halobatrachus didactylus* (Pisces: Batrachoididae). *Acta Histochem.* **113**, 256–261 (2011). [https://doi.org/10.1016/j.acthis.2009.10.007](https://doi.org/10.1016/j.acthis.2009.10.007)

101. Lazarow, A., & Cooperstein, S. J. Studies on the isolated islet tissue of fish. I. the cytochrome oxidase and succinic dehydrogenase contents of normal toadfish (*Opsanus tau*). *Biol. Bull.* **100**, 191-198 (1951). <https://doi.org/10.2307/1538530>

102. Kelley, K. M. Experimental diabetes mellitus in a teleost fish. I. Effect of complete isletectomy and subsequent hormonal treatment on metabolism in the goby, *Gillichthys mirabilis. Endocrinology* **132**, 2689-2695 (1993). https://doi.org/[10.1210/endo.132.6.8504768](https://doi.org/10.1210/endo.132.6.8504768)

103. Patent, G. J., Kechele, P. O., & Tomichek Carrano, V. Nonconventional innervation of the pancreatic islets of the teleost fish, *Gillichthys mirabilis*. *Cell Tissue Res*. **191**, 305-315 (1978). <https://doi.org/10.1007/BF00222426>

104. Kotaki, A. Studies on Insulin. III. On the structure of tha alanyl chain of Bonito insulin*. J. Biochem.* **51**, 301-309 (1962).

105. Suyehiro, Y. On the pancreas and the islets of Langerhans of *Scomber*. *Nippon Suisan Gakkaishi* **12**, 157-159 (1944). <https://doi.org/10.2331/suisan.12.157>

106. Navarro, I., Gutiérrez, J., Caixach, J., Rivera, J., & Planas, J. Isolation and primary structure of glucagon from the endocrine pancreas of *Thunnus obesus. Gen. Comp. Endocrinol.* **83**, 227-232 (1991). [https://doi.org/10.1016/0016-6480(91)90025-2](https://doi.org/10.1016/0016-6480(91)90025-2)

107. Planas, J., & Lluch, M. Accion hiperglucemiante de los extractos de nodulos pancreaticos endocrinos en el atun. *Rev. Esp. Fisiol.* **12**, 295-300 (1956).

108. Planas, J., & Garcia, F. New data regarding the pancreatic islets in the tunny-fish. *Acta Anat.* **57**, 185-191 (1964). <https://doi.org/10.1159/000142548>

109. Khanna, S. S., & Singh T. Histology of the principal islets of a fresh water teleost, *Channa punctatus* (Bloch). *Acta Anat.* **78**, 99–106 (1971). <https://doi.org/10.1159/000143580>

110. [Schrezenmeir, J](http://www.ncbi.nlm.nih.gov/pubmed/?term=Schrezenmeir%20J%5BAuthor%5D&cauthor=true&cauthor_uid=8184466). *et al*. Effect of microencapsulation on oxygen distribution in islets organs. *Transplantation* **57**, 1308-1314 (1994). https://doi.org/[10.1097/00007890-199405150-00003](https://doi.org/10.1097/00007890-199405150-00003)

111. Watari, N., Tsukagoshi, N., & Honma, Y. The correlative light and electron microscopy of the islets of Langerhans in some lower vertebrates. *Arch. Histol. Jpn.* **31**, 371-392 (1970). <https://doi.org/10.1679/aohc1950.31.371>

112. Langdon, J.S. [Diseases of mahi mahi or common dolphin fish, *Coryphaena hippurus* in Australia](https://researchlibrary.agric.wa.gov.au/cgi/viewcontent.cgi?article=1006&context=fr_frb). *Fish. Res. Bull. Fish. West. Austr.* **29**, 1-17 (1991).

113. Honma, Y., & Yoshie, S. Histological observations on some of the endocrine glands in the remora, *Echeneis naucrates* L., caught off the coast of Sado Island in the Japan Sea. *Arch. Histol. Jpn.* **37**, 261-273 (1974). <https://doi.org/10.1679/aohc1950.37.261>

114. Faulk, C. K., Benninghoff, A. D., & Holt, G. J. Ontogeny of the gastrointestinal tract and selected digestive enzymes in cobia *Rachycentron canadum* (L.) *J. Fish Biol.* **70**, 567–583 (2007). <https://doi.org/10.1111/j.1095-8649.2007.01330.x>

115. Hirano, S., & Honma, Y. Cytological studies on the endocrine pancreas of fishes and cyclostomes with special regard to the islet cells of the sailfish*, Istiophorus platypterus* (Shaw et Nodder). *Annual Report of the Sado Marine Biological Station, Niigata University* **1**, 1-15 (1971).

116. Yoshida, K., Iwanaga, T., & Fujita, T. Gastro-entero-pancreatic (GEP) endocrine system of the flatfish, *Paralichtys olivaceus*: an immunocytochemical study. *Arch. Histol. Jpn.* **46**, 259-266 (1983).

117. Andoh, T., & Nagasawa, H. Purification and structural determination of insulins, glucagons and somatostatin from stone flounder, *Kareius bicoloratus. Zool. Sci.* **15**, 939-943 (1998). <https://doi.org/10.2108/zsj.15.939>

118. Thomas, N. W. Observations on the cell types present in the principal islet of the dab *Limanda limanda*. *Gen. Comp. Endocrinol.* **26**, 496-503 (1975). [https://doi.org/10.1016/0016-6480(75)90172-0](https://doi.org/10.1016/0016-6480(75)90172-0)

119. Suehiro, M. Historical review of insulin and its preparations in pharmacopoeia (3). Fish insulins. *Yakushigaku Zasshi* **27**, 32-39 (1992).

120. Conlon, J. M., Falkmer, S., & Thim, L. Primary structures of three fragments of proglucagon from the pancreatic islets of the daddy Sculpin (*Cottus scorpius*). *Eur. J. Biochem.* **164**, 117-122 (1987). <https://doi.org/10.1111/j.1432-1033.1987.tb11001.x>

121. Maske, H., & Munk, K. Über die Verteilung von Insulin und Zink in verscheidenen Zellbestandteilen der Rieseninseln bei Flundern und Schollen (Pleuronectiden). *Z. Naturforsch.* **11**, 407-415 (1956).

122. Andoh, T. Plasma insulin levels are regulated by release, rather than transcription or translation, in barfin flounder, *Verasper moseri. Comp. Biochem. Physiol. Part A Mol. Integr. Physiol.* **184**, 27–33 (2015). [https://doi.org/10.1016/j.cbpa.2015.01.020](https://doi.org/10.1016/j.cbpa.2015.01.020)

123. Berwert, L., Segner, H., & Reinecke M. Ontogeny of IGF-1 and the classical islet hormones in the turbot, *Scophthalmus maximus*. *Peptides* **16**, 113-122 (1995). [https://doi.org/10.1016/0196-9781(94)00161-X](https://doi.org/10.1016/0196-9781(94)00161-X)

124. Padrós, F., Villalta, M., Gisbert, E., & Estévez, A. Morphological and histological study of larval development of the Senegal sole *Solea senegalensis*: an integrative study. *J. Fish Biol.* **79**, 3–32 (2011). <https://doi.org/10.1111/j.1095-8649.2011.02942.x>

125. Sarasquete, C., Gisbert E., & Ortiz-Delgado, J. B. Embryonic and larval ontogeny of the Senegalese sole, *Solea senegalensis*: Normal patterns and pathological alterations. In *The Biology of Sole* (eds. Muñoz-Cueto, J. A., Mañanos, E., & Sánchez-Vázquez, F. J.) 37p (CRC Press, 2019).

126. Langer, M., Van Noorden, S., Polak, J. M., & Pearse, A. G. E. Peptide hormone-like immunoreactivity in the gastrointestinal tract and endocrine pancreas of eleven teleost species. *Cell Tissue Res.* **199**, 493-508 (1979). <https://doi.org/10.1007/BF00236085>

127. Assouline, B., Nguyen, V., Mahé, S., Bourrat, F., & Scharfmann, R. Development of the pancreas in medaka. *Mech. Dev.* **117**, 299-303 (2002). [https://doi.org/10.1016/S0925-4773(02)00190-9](https://doi.org/10.1016/S0925-4773(02)00190-9)
128. Suyehiro, Y. On the digestive system and the feeding habit of the Japanese flying fish, *Cypselurus agoo*. *Bull. Jap. Soc. Sci. Fish* **4**, 37-44 (1935).
129. Bullock, W. L. The intestinal histology of the mosquito fish, *Gambusia affinis* (Baird and Girard). *Acta Zool*. **48**, 1-17 (1967). <https://doi.org/10.1111/j.1463-6395.1967.tb00129.x>
130. Klein, C., & Lange, R. H. Principal cell types in the pancreatic islet of a teleost fish, *Xiphophorus helleri* H. *Cell Tissue Res.* **176**, 529-551 (1977). <https://doi.org/10.1007/BF00231406>

131. Klein, C., & Van Noorden, S. Pancreatic polypeptide (PP)- and glucagon cells in the pancreatic islet of *Xiphophorus helleri* H. (Teleostei). *Cell Tissue Res.* **205**, 187-198 (1980). <https://doi.org/10.1007/BF00234679>

132. Lozano, M. T., & Agulleiro, B. Immunocytochemical and ultrastructural study of the endocrine pancreas of *Mugil auratus* and *Mugil saliens* L. (Teleostei). *J. Submicrosc. Cytol.* **18**, 85-98 (1986).

133. Maglio, M., & Putti, R. Morphological basis of the interaction between endocrine cell types in the endocrine cell types in the pancreatic islets of the teleost, *Blennius gatturoggine. Tissue Cell* **30**, 672-683 (1998).

134. Putti, R., Maglio, M., & Odierna, G. An immunocytochemical study of intrapancreatic ganglia, nerve fibres and neuroglandular junctions in Brockmann bodies of the tompot blenny (*Blennius gattoruggine*), a marine teleost. *Histochem. J.* **32**, 607-616 (2000). <https://doi.org/10.1023/A:1026740606290>

135. Bowie, D. J. Cytological studies of the islets of Langerhans in a teleost, *Neomaenis griseus*. *Anat. Rec.* **29**, 57-73 (1925).

136. Tong, C.-M., Chen, N.-S.., Ji, Z.-Y., Xu, X.-T., & Gou, S.-P. The histological observation of the pancreas of largemouth bass, *Micropterus salmoides*. *Journal of Shanghai Ocean University* **23**, 814-819 (2014).

137. Carrillo, M., Zanuy, S., Duve, H., & Thorpe A. Identification of hormone-producing cells of the endocrine pancreas of the sea bass, *Dicentrarchus labrax*, by ultrastructural immunocytochemistry. *Gen. Comp. Endocrinol*. **61**, 287-301 (1986). [https://doi.org/10.1016/0016-6480(86)90206-6](https://doi.org/10.1016/0016-6480(86)90206-6)

138. Agulleiro, B., García Hernández, M. P., & Lozano, M. T. Ontogeny of the endocrine pancreas in sea bass (*Dicentrarchus labrax*): an ultrastructural study. II. The big and secondary islets. *Cell Tissue Res.* **276**, 323–331 (1994). <https://doi.org/10.1007/BF00306117>

139. Groman, D. B. *Histology of the striped bass*. (American Fisheries Society, 1982).

140. Al-Hussaini, A. H. The anatomy and histology of the alimentary tract of the coral feeding fish *Scarus sordidus* (Klunz). *Bulletin de l’Institute d’Égypte* **27**, 349–377 (1945).

141. Abad, M. E., [Agulleiro](http://www.sciencedirect.com/science/article/pii/0016648086900213), B., & Rombout, J. H. W. M. An immunocytochemical and ultrastructural study of the endocrine pancreas of Sparus auratus L. (Teleostei). *Gen. Comp. Endocrinol.* **64**, 1­­–12 (1986). [https://doi.org/10.1016/0016-6480(86)90021-3](https://doi.org/10.1016/0016-6480(86)90021-3)

142. Navarro, M. H., Lozano, M. T., & Agulleiro, B. Ontogeny of the endocrine pancreatic cells of the gilthead sea bream, *Sparus aurata* (Teleost). *Gen. Comp. Endocrinol.* **148**, 213-226 (2006). https://doi.org/[10.1016/j.ygcen.2006.03.007](https://doi.org/10.1016/j.ygcen.2006.03.007)

143. Johnson, D. E. et al. Immunohistochemical localization of somatostatin, insulin and glucagon in the principal islets of the anglerfish (*Lophius americanus*) and the channel catfish (*Ictalurus punctata*). *Am. J. Anat.* [**147**,](http://onlinelibrary.wiley.com/doi/10.1002/aja.v147:1/issuetoc)119–124 (1976). <https://doi.org/10.1002/aja.1001470112>

144. Chiba, A., Yoshie, S., & Honma, Y. Histological observations of some organs in the triggerfish, *Canthidermis rotundatus*, stranded on the coast of Niigata facing the Japan Sea. *Jpn. J. Ichthyol.* **22**, 212-220 (1976).

145. Chiba, A., & Honma, Y. Histological observations of some organs in the porcupine fish *Diodon holacanthus*, stranded in Niigata on the coast of Japan Sea. *Jpn. J. Ichthyol.* **28**, 287-294 (1981).

146. Endo, Y., [Chiba, A](http://www.ncbi.nlm.nih.gov/pubmed/?term=Chiba%20A%5BAuthor%5D&cauthor=true&cauthor_uid=1678152)., & Honma, Y. Exocytotic release of neurotransmitters from nerve fibers in the endocrine pancreas (Brockmann body) of the teleost, *Takifugu niphobles. Neurosci. Lett.* **123**, 60-62 (1991). [https://doi.org/10.1016/0304-3940(91)90371-Y](https://doi.org/10.1016/0304-3940(91)90371-Y)

147. Kobayashi, K., [Shibasaki, S](http://www.ncbi.nlm.nih.gov/pubmed/?term=Shibasaki%20S%5BAuthor%5D&cauthor=true&cauthor_uid=793720)., & [Takahashi, Y.](http://www.ncbi.nlm.nih.gov/pubmed/?term=Takahashi%20Y%5BAuthor%5D&cauthor=true&cauthor_uid=793720) Light and electron microscopic study on the endocrine cells of the pancreas in a marine teleost, *Fugu rubripes rubripes*. *Cell Tissue Res.* **174**, 161-182 (1976). <https://doi.org/10.1007/BF00222157>

148. Western, J. R. H. Studies on the diet, feeding mechanism and alimentary tract in two closely related teleosts, the freshwater *Cottus gobio* L. and the marine *Parenophrys bubalis* Euphrasen. *Acta Zool.* **50**, 185–205 (1969). <https://doi.org/10.1111/j.1463-6395.1969.tb00540.x>

149. Hellman, B., & Larsson, S. The glucose metabolism in the islets of Langerhans. I. in vitro studies of the fate of uniformly ^14^C-labelled glucose and fructose in *Cottus quadricornis* L. *Acta Endocrinol.* **38**, 303-314 (1961). <https://doi.org/10.1530/acta.0.0380303>

150. Cutfield, J. F., Cutfield, S. M., Carne, A., Emdin, S. O., & Falkmer, S. The isolation, purification and amino-acid sequence of insulin from the teleost fish *Cottus scorpius* (daddy sculpin). *Eur. J. Biochem.* **158**, 117- 123 (1986). <https://doi.org/10.1111/j.1432-1033.1986.tb09728.x>

151. [Syed Ali, S](http://www.ncbi.nlm.nih.gov/pubmed/?term=Syed%20Ali%20S%5BAuthor%5D&cauthor=true&cauthor_uid=3909984). Microvasculature of the principal islets in the scorpion fish, *Myoxocephalus scorpius. Arch. Histol. Jpn*. **48**, 363-371 (1985). <https://doi.org/10.1679/aohc.48.363>

152. Berkeley, C. The distribution of pentose compounds in the pancreatic tissues of the ling cod (*Ophiodon elongatus*, Girard). *J. Biol. Chem*. **58**, 611-616 (1923).

153. Mosca, L. An experimental study of the cytology of pancreatic islets. *Q. J. Exp. Physiol. Cogn. Med. Sci.* **42**, 49–55 (1957). https://doi.org/[10.1113/expphysiol.1957.sp001242](https://doi.org/10.1113/expphysiol.1957.sp001242)

154. Hassan, A. A. Anatomy and Histology of the digestive system of the carnivorous fish, the brown-spotted grouper, *Epinephelus chlorostigma* (Pisces; Serranidae) from the Red Sea. *Life Sci.* 10, 2149- 2164 (2013).

155. Tagliafierro, G., Carlini, M., Faraldi, G., & Gallus, L. The neuroendocrine system in the intestinal tract and pancreas of Antarctic fish. In *Fishes of Antarctica. A biological overview* (eds. di Prisco, G., Pisano, Ε., & Clarke, A) 247-256 (Springer-Verlag, Milano, 1998). <https://doi.org/10.1007/978-88-470-2157-0_22>

156. Eastman, J. T., & DeVries, A. L. Morphology of the digestive system of Antarctic nototheniid fishes. *Polar Biol.* **17**, 1-13 (1997). <https://doi.org/10.1007/s003000050098>

157. Bachle, L. A., Smith, D. D., & Petzel, D. Isolation and characterization of insulin from the Brockmann body of *Dissostichus mawsoni*, an Antarctic teleost fish. *J. Pept. Res.* [**56**,](http://onlinelibrary.wiley.com/doi/10.1111/jpp.2000.56.issue-1/issuetoc) 47–54 (2000). <https://doi.org/10.1034/j.1399-3011.2000.00742.x>
